# Treating Low-Grade Myxofibrosarcoma With Stereotactic Body Radiation Therapy Using CyberKnife®

**DOI:** 10.7759/cureus.16393

**Published:** 2021-07-14

**Authors:** Shinichiro Mizumatsu, Hiroshi Ryu, Kei Nomura, Satoshi Yoshikawa, Norio Inoue

**Affiliations:** 1 CyberKnife Center, Aoyama General Hospital, Toyokawa, JPN; 2 Cerebrospinal Center, Aoyama General Hospital, Toyokawa, JPN; 3 Recovery Rehabilitation Center, Aoyama General Hospital, Toyokawa, JPN

**Keywords:** myxofibrosarcoma, stereotactic body radiation therapy, cyberknife, adjuvant therapy, hypofractionated radiation therapy, soft tissue sarcoma, radiation therapy, bone and soft tissue tumors, low-grade myxofibrosarcoma

## Abstract

Myxofibrosarcoma (MFS) is one of the most common soft tissue sarcomas. Low-grade MFS has a high local recurrence rate, similar to that of high-grade MFS. Hence, appropriate adjuvant therapy is required to control low-grade MFS. In this report, we present a case in which recurrent low-grade MFS was successfully treated with stereotactic body radiation therapy (SBRT) using CyberKnife® (CK) (Accuray Incorporated, Sunnyvale, CA). A 76-year-old man underwent SBRT using CK for recurrent low-grade MFS in the right posterior chest wall after undergoing resection and skin grafting four and three times, respectively. We planned CK treatment separately for each in two parts. For the lesion on the scapula side, the target volume was 109 cm^3 ^and the total prescribed dose was 34.6 Gy, while the lesion on the spinal side had a target volume of 72 cm^3^ and a total prescribed dose of 36 Gy, both in five fractions. Each SBRT was performed on alternate days in a span of 14 days. The tumors gradually reduced in size with tolerable levels of toxicity. SBRT using CK could be a safe and effective adjuvant therapy for low-grade MFS.

## Introduction

Myxofibrosarcoma (MFS) is one of the most common soft tissue sarcomas affecting middle-aged and elderly people. The optimal treatment for MFS is radical surgical resection, including a wide margin of adjacent disease-free tissue. However, this treatment method may lead to a considerably reduced quality of life. Radiation therapy (RT) has been described as a definitive treatment for unresectable MFS. Furthermore, a novel advanced technique, stereotactic body radiation therapy (SBRT), allows for the delivery of a higher dose of radiation to the tumor while reducing the quantity of irradiation to the surrounding normal tissue. Herein we present a case in which postoperative recurrent low-grade MFS was successfully treated with SBRT using CyberKnife® (CK) (Accuray Incorporated, Sunnyvale, CA).

## Case presentation

**Figure 1:**
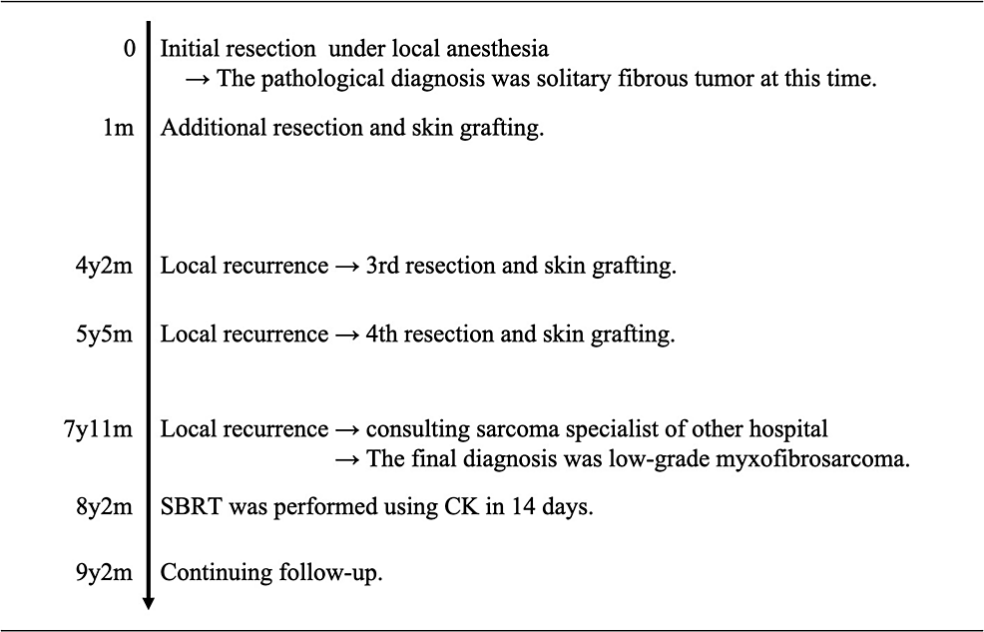
Clinical course SBRT: stereotactic body radiation therapy: CK: CyberKnife

The patient was a 76-year-old man with a past history of ischemic heart disease (on antiplatelet drug), aortic regurgitation after bio-valve replacement, and pituitary dysfunction. His blood type was type AB, Rh (−). In 2012, he presented with an approximately one-year history of a gradually increasing painless mass (9 x 6 cm) in the right posterior chest wall. A dermatologist at the first hospital performed the initial surgery on him without sufficient preoperative evaluation by imaging. The initial pathological diagnosis was a solitary fibrous tumor. In total, he underwent resection and skin grafting four and three times, respectively, in order to treat a tumor on the right posterior chest wall (Figure 1).

**Figure 2:**
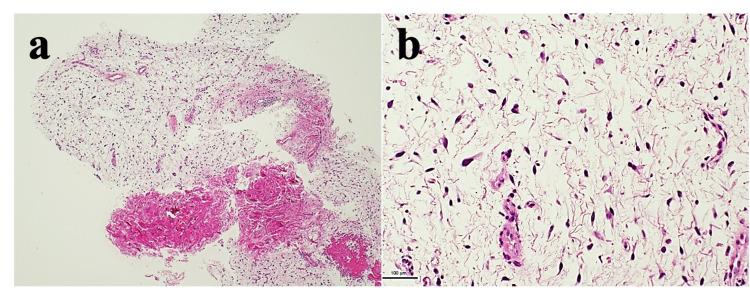
Histopathological findings (a). Low power field, x 2 (b). High power field, x 20 Hematoxylin & eosin staining of the recurrent tumor by needle biopsy. Spindle-shaped cells and curvilinear thin-walled blood vessels in a myxoid background in low cellularity tumor. The tumor cells showing no severe atypia, no pleomorphism, and few mitoses

The tumor recurred locally 30 months after the fourth resection. He was referred to the second hospital and seen by a sarcoma specialist. The final pathological diagnosis was low-grade MFS with 5% of Ki-67 labeling index (Figure 2).

The patient was advised to undergo treatment using CK at our institution because the referring specialist considered the tumor to be unresectable. 

**Figure 3:**
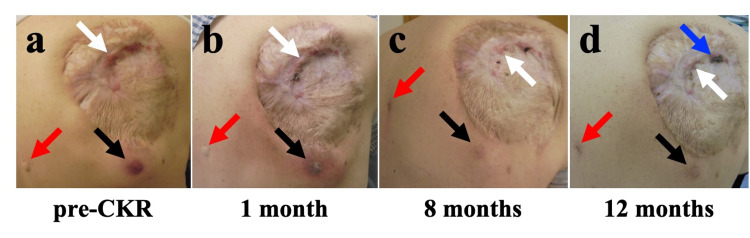
Time course of skin findings on photography The images show changes in the right-back chest wall skin after CKR. Subcutaneous lesions also improved after CKR (a). Three lesions (three arrows) and two areas of redness (black and white arrows) (b). One lesion reduced in terms of the surgical scar (white arrow). The other lesion increased surrounding redness (black arrow). Another lesion had no change (red arrow) (c). Two lesions improved (black and white arrows). The other lesion appeared with redness (red arrow) (d). Two lesions maintained improvement (black and red arrow). The other lesion in the surgical scar did not increase (white arrow) but a blood scab occurred in part (blue arrow) CKR: CyberKnife radiotherapy

At the first visit, he presented with a subcutaneous tumor in the right posterior chest wall and felt heavy, and faced difficulty raising his right upper limb without pain. Three lesions were found on his back. The skin on the tumor was thin and fragile, but not perforated yet (Figure 3).

**Figure 4:**
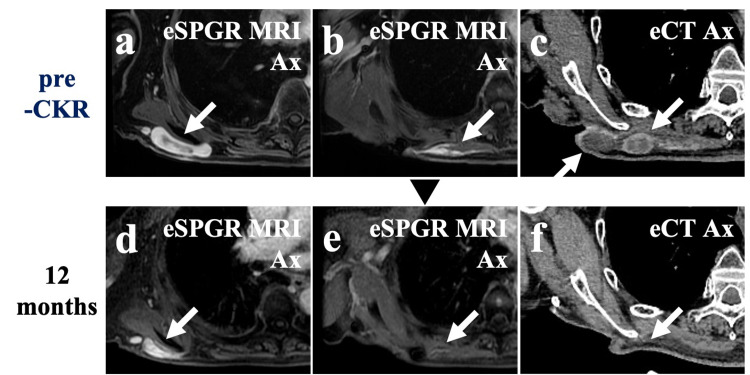
MRI and CT before and after CKR (a), (b), and (c) are pre-CKR images. (d), (e), and (f) are images 12 months after CKR. (a), (b), (d), and (e) are enhanced SPGR MRIs. (c) and (f) are enhanced CT (a). Enhanced lesions (white arrow) (b). The tail sign along the fascia (white arrow) (c). Nodular lesions with the enhanced wall (d). The enhanced lesion has decreased (e). The enhancement of the tail sign has disappeared (f). The enhanced lesions have disappeared MRI: magnetic resonance imaging; CT: computed tomography; CKR: CyberKnife radiotherapy; eSPGR: enhanced spoiled gradient-recalled; eCT: enhanced CT; Ax: axial view

Plain and enhanced CT, plain and enhanced MRI, and 2-deoxy-2-[18F] fluoro-D-glucose (FDG)-positron emission tomography (PET)/CT were performed as pretreatment examinations. CT and MRI revealed contrast-enhanced multiple nodules in the right posterior chest wall with irregular surfaces (Figure 4).

**Figure 5:**
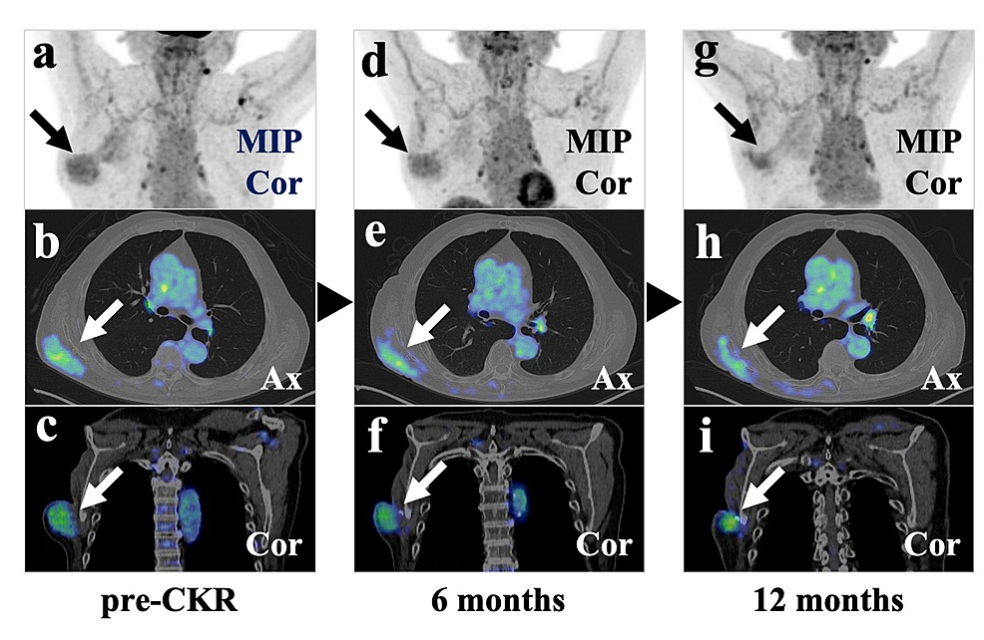
Time course of FDG-PET/CT (a), (b), and (c). A scapula side lesion with relatively high FDG uptake in the right posterior chest wall before CKR (black and white arrows) (d), (e), and (f). The lesion FDG uptake reduced six months after CKR (black and white arrows) (g), (h), and (i). The lesion FDG uptake reduced further 12 months after CKR (black and white arrows) CKR: CyberKnife radiotherapy; FDG: 2-deoxy-2-[18F] fluoro-D-glucose; PET: positron emission tomography; CT: computed tomography; MIP: maximum intensity projection; Cor: coronal view; Ax: axial view

Enhanced MRI revealed a tail sign, i.e., a thick fascial enhancement extending from the tumor margin, in the right erector spinae muscle (Figure 4). High FDG uptake in the lesion was observed on PET/CT (Figure 5).

No other abnormal findings, such as suspected metastasis, were observed.

**Figure 6:**
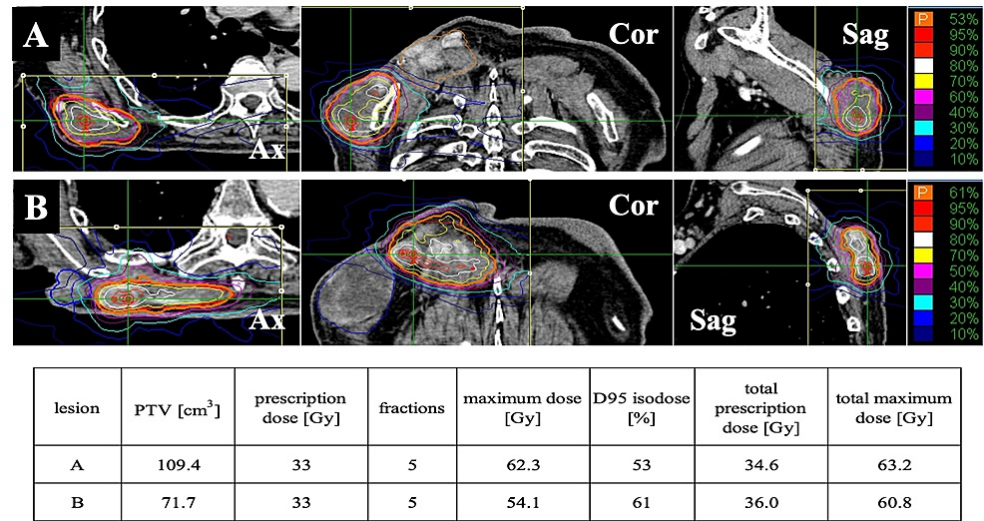
CyberKnife radiotherapy plan using MultiPlan (A). The plan for scapula side lesion (B). The plan for spinal side lesion Table – CKR: CyberKnife radiotherapy; PTV: planning target volume; total dose: plan A + B Ax: axial view; Cor: coronal view; Sag: sagittal view; PTV: planning target volume

We planned the CK treatment separately for each lesion in two parts because of the irregularity in the shape of the lesions. SBRT was performed using CK G4® (Accuray) system with MultiPlan® (Accuray). The target lesions were tracked with the Xsight® (Accuray) spine tracking system. The gross tumor volume (GTV) was contoured using planning CT with MRI and FDG-PET/CT. We decided that the planning target volume (PTV) and GTV were the same. For the lesion on the scapula side, the PTV was 109 cm^3^ and the total prescribed dose was 34.6 Gy, while the lesion on the spinal side had the PTV of 72 cm^3^ and a total prescribed dose of 36 Gy, both in five fractions (Figure 6). In the right lung, the volume receiving over 20 Gy was 0.6% and the total maximum dose was 28.6 Gy. The total maximum dose on the skin surface was 39.5 Gy and that in the spinal cord was 14.1 Gy.

Each SBRT was performed on alternate days in a span of 14 days. The patient felt an improvement in his right upper limb as it got lighter during SBRT. There was no severe adverse event during and after SBRT. The tumor gradually decreased in size and the range of movement of his right upper limb expanded after SBRT. PET performed six and 12 months after SBRT showed a decrease in the uptake of FDG by the tumor (Figure 5). CT and MRI revealed decreased size of the tumor (Figure 4). All image examinations showed residual tumor at the boundary with the resected area (Figures 4, 5). Redness and swelling occurred around the lesions away from the resected area but recovered spontaneously (Figure 2). He occasionally had blood oozing due to rubbing the fragile skin in the resected area (Figure 3). CT revealed transient inflammatory findings in the right lung without any symptoms. PET/CT performed six and 12 months after SBRT revealed no FDG uptake in the right lung findings.

One year after SBRT, the patient continues to lead a normal life. We would like to perform additional treatment using CK when the residual tumor has increased in size.

## Discussion

MFS is a fibroblast-derived soft tissue sarcoma that commonly occurs subcutaneously in the limbs of elderly people. MFS accounts for approximately 9% of all soft tissue malignant tumors [1]. In general, MFS is clinically characterized as a slow-growing, partially nodular, and painless tumor. MFS is classified into three grades: low, intermediate, and high. Low-grade MFS is unlikely to cause distant metastasis, but its local recurrence rate is as high as 50-60%, which is similar to that of high-grade MFS [2,3]. The grade of MFS tends to progressively increase with recurrence [2,3].

Complete surgical resection at the time of primary tumor presentation remains the most effective therapy for MFS. It is generally believed that resection of at least 2 cm around the tumor is needed to ensure a negative surgical margin [4]. The tumor eventually becomes inoperable due to frequent repeated local recurrences and subsequent surgeries. The effect of chemotherapy is unclear; however, it is commonly used in patients with MFS with distant metastasis.

RT is a reasonable option for local treatment of tumors in situations where the effect of chemotherapy is uncertain. Sufficient doses of radiation may not be administered for the tumor of the trunk due to difficulty in surgery. Consequently, RT is mostly performed with a palliative aim, such as pain relief. Callegaro et al. have reported that RT was associated with a better local outcome, especially in myxoid liposarcoma, vascular sarcoma, and MFS, without being associated with survival [5]. However, no randomized controlled trials have specifically evaluated the effect of radiotherapy on MFS. The advantage of treating tumors with SBRT over conventional radiation therapy (CRT) is that SBRT precisely irradiates tumors while allowing a tight margin of surrounding normal tissues. Furthermore, the therapeutic effect of SBRT for MFS in one to five fractions may be greater than that with CRT because the α/β ratio of soft tissue sarcoma is known to be relatively low [6].

CK is an SBRT device with image guidance, and it consists of a robot arm, linear accelerator, and target tracking system. This system can irradiate a target with less damage to proximal organs by moving the robot arm, which has a wide range of motion. SBRT using CK is less invasive as compared with standard surgery and has a shorter treatment period and higher efficacy than CRT. Paik et al. have reported that SBRT using CK for soft tissue tumors yielded acceptable results with irradiation doses ranging from 20 to 48 Gy in one to five fractions [7]. Additionally, Wang et al. have reported that an unresectable chest-wall malignant fibrous histiocytoma (MFH), which received CK irradiation at a dose of 75 Gy in five fractions over five days, demonstrated gradual shrinking of the tumor with tolerable toxicity following the treatment [8]. However, there is no consensus on whether and when radiotherapy should be used in the management of MFS. Furthermore, the tolerable dose of fragile skin after repeated resection is unknown. Zaorsky et al. [9] have reported good cosmesis in 80% of patients with skin cancer after RT under a biologically effective dose (BED) of 100 Gy (α/β=3) in a meta-analysis. This BED dose corresponds to 50 Gy in 15 fractions, 36.7 Gy in seven fractions, and 35 Gy in five fractions as a treatment radiation dose. It has been reported that protocols with irradiation every alternate day resulted in the reduction of adverse events, such as rectal toxicity in prostate cancer, carotid blowout syndrome in recurrent head and neck cancer, and general fatigue in hepatocellular carcinoma after treatment with SBRT [10-12]. Kubicek et al. [13] have reported that surgical complications and control rates were satisfactory with a regimen of 35-40 Gy in five fractions every alternate day as a preoperative RT for soft tissue sarcoma. There are also two reports stating that using hypofractionated RT as preoperative RT was a safer alternative to CRT in wound complications after surgical resection for soft tissue sarcoma [14,15]. Parsai et al. [14] used a regimen of 30 Gy in five fractions (range: 27.5-40 Gy) on consecutive days, whereas Allen et al. [15] used 28 Gy in eight fractions with intensity-modulated radiation therapy.

We treated the two lesions by alternatively irradiating them every other day to reduce damage to the fragile skin. In the present case, CK radiotherapy was delivered at a prescribed dose of 33 Gy in five fractions, totaling 35-36 Gy over 14 days. Following the treatment, the tumor gradually reduced in size with tolerable levels of toxicity.

## Conclusions

Appropriate adjuvant therapy is needed to control low-grade MFS due to the high chances of frequent local recurrence. Our case report demonstrates that SBRT using CK might be safe and effective for managing unresectable low-grade MFS. Studies with longer follow-ups are required to further confirm the efficacy and safety of this treatment modality.
